# Facts and Theories about the Recent Outbreak of Asiatic Cholera

**Published:** 1874-01

**Authors:** John C. Peters

**Affiliations:** New York


*Facts and Theories about the Recent Outbreak of Asiatic Cholera.* By JOHN C. PETERS, M.D., of New York.

By a true theory we mean a legitimate deduction from facts, not contradicted by equally numerous and well-substantiated facts. An hypothesis is a very different thing.

We all know how the great outbreak of cholera, in 1865, was carried from Alexandria, in Egypt, by steamships to all parts of Europe, and notably to the Black-Sea ports of Russia, and thence over the whole Russian Empire. Next came the pestilence at Hurdwar, in Northern India, in 1867, which was carried through Northern Persia to the Caspian Sea, and thence across to Russia. Then came the virulent epidemic of 1869 in the Punjab, or extreme northwestern province of India, which was also carried through Northern Persia to Astrabad, a port on the southeastern corner of the Caspian Sea, and from there again over to Russia. These successive outbreaks in Persia and Russia have led to the belief by Dr. Tholazon, of Persia, and Dr. Pelikan, of Russia, that cholera had become naturalized in these two countries. But the annual reports of the sanitary commissioner with the government of India, especially that of 1869 (see p. 220), prove the above facts. Inspector-General Murray has also established the former in opposition to the latter views. The most interesting and important town in Northern Persia, in connection with conveyance of cholera to Europe, is the holy city of Meshed, situated about two-thirds of the way from India toward the Caspian Sea. This city is so holy that no person of any sect called Mohammedan has ever dared to commit the impiety of firing a hostile shot at its walls. For eight months in the year all the roads to and from Meshed are thronged with pilgrims. It is calculated that nearly sixty thousand come up from India, Cabool, and Afghanistan, often bringing cholera with them; and an equal number coming from Turkey in Asia, and the countries between the Black and Caspian Seas, frequently carry it back with them. It is not a little singular, that, among the first towns attacked in Russia, in 1870 or '71, was the holy city of Kiev on the right bank of the Dnieper, in the southwestern portion of Russia, about one hundred miles north of Odessa, on the Black Sea, and four hundred miles southwest of Moscow. Out of a population of seventy thousand it was losing nearer one hundred than fifty a day from cholera, when almost all the rest of Russia was free from it. About fifty thousand pilgrims come to Kiev every year to visit the relics of the one hundred and ten martyrs to the old Russian faith. It is adorned with a large number of magnificent churches, the great cathedral of St. Sophia, and the palace of the Greek metropolitan. The glittering gilt, green, and varied-colored cupolas on the numerous eminences present a magnificent spectacle from a distance. It is the port and capital of the Ukraine, and is near Poland, Galicia, and Moldavia. It has been the capital of the princes of Novgorod since the ninth century, and Panslavic patriots still look to it as their natural capital. It is magnificent but filthy. The drains, privy-vaults, and out-houses of Kiev are in the most primitive and disgusting condition, and on every hot day a shocking odor ascends from that otherwise glorious town. A distinguished architect once boasted that he had built a thousand houses in Kiev but not one water-closet. It always requires as much sanitary inspection as Meshed or Mecca. The mortality in 1871 among the pilgrims and in the monasteries was very great.

From Kiev cholera spread easily to Poland and down the river Vistula to the Baltic. Last year it had obtained a lodgment not only in Hungary and Poland, but in the Baltic towns of Konigsberg, Elbing, Dantzic, Stettin, and also in Hamburg and Bremen. When cholera commenced in New Orleans, on Feb. 9, 1873, of course the steamers from Northern Germany, which touch at Havre, were first suspected, especially as the first death from cholera in New Orleans was that of a Prussian aged 56, and the next that of a Frenchman.* But so many rumors were raised about vessels from Odessa, the Mediterranean, and the Baltic, that attention was quite diverted from the North-Sea steamers. But the similarity to the outbreak in 1848 and '49 was too great to pass unnoticed. 1. In 1848 the ship Guttemberg sailed from Hamburg, where cholera had prevailed from Sept. 7th, with two hundred and fifty steerage passengers; had several deaths from cholera while still in the Elbe, and arrived at New Orleans on Dec. 6th, after a passage of fifty-five days. She had no more deaths from cholera, but some from diarrhœa and dysentery, and doubtless had infected clothing on board. 2. The bark Callao, from Bremen, with one hundred and fifty emigrants, had eighteen deaths from cholera, the last one on Nov. 8th, doubtless also with infected articles on board. 3. The ship Swanton, from Havre, had seventeen deaths from cholera, arrived

* These proved to be old residents.

at New Orleans on Dec. 11, 1848, and on the 12th a woman was carried from the ship to the Charity Hospital in a state of complete cholera-collapse. One case was traced to the Guttemberg, five or six others to passengers from the ship Swanton, who had not been subjected to quarantine, but had scattered all over the city. Cholera commenced in December, 1848, in New Orleans, almost immediately after the arrival of the Swanton, which is quite unusual in large cities, in which the commencement of an epidemic of cholera is generally very slow. It was suspected, but never could be proved, that other cholera vessels had arrived previous to the Guttemberg, Callao, and Swanton. For, it may be assumed as a fact, when cholera breaks out suddenly and violently in a large town, that many cases of choleraic diarrhœa, cholerine, choleraic cholera-morbus, have been overlcoked. It was decided as early as 1848 and '49, in England, that "the popular notion that cholera is sudden in its invasions of large towns or districts is as unfounded as the formerly prevalent opinion that it is always sudden in its attack in individuals."

In 1848 and 1849, in London, Edinburgh, Glasgow, Plymouth, Dundee, Bristol, Liverpool, Hull, and almost every large city in Great Britain in which the first cases were accurately observed, the initial case or cases preceded the others by from one to several weeks (see Dr. Sutherland's report to Parliament, pages 13—17). The first attacks were isolated, and occurred at considerable distances apart as to place, and intervals as to time. This mode of outbreak may be regarded as one of the laws of an epidemic of cholera. As the disease is generally brought by steerage passengers, immigrants, and the poorer class of travelers, the initial cases in large towns generally occur in the low haunts and outskirts. They are widely separated, as if springing from distinct sources of infection, and often occur among old inhabitants, because clothing or fomites have been brought to them, or because they have visited or been visited by some one with choleraic diarrhœa, or cholerine. However this may be, the New Orleans authorities have not yet succeeded in tracing the direct importation of the disease. "From December 1, 1872, to May 1, 1873, no vessel from Odessa, or any other port on the Black Sea, came to New Orleans. From January 1 to May 1, 1873, no vessel from the Baltic came to New Orleans. In January, 1873, *passengers* arrived only from Hamburg, Bremen, and Liverpool. In February, only from Bremen, Port Simon, and Liverpool. In March, from Hamburg, Liverpool, Palermo, Bremen, and Mexico. In April, only from Liverpool, Bremen, Hamburg, and Port Simon. It is claimed that none of these vessels, or any other, had deaths or sickness from cholera. Only two sailors were attacked with cholera, and only one died. Both were from the British ship Belgravia, which had no passengers, was some fifty days on the voyage, and they were taken ill ten days at least after arrival, when Asiatic cholera was already prevailing, viz., about April 14th and 16th. As the disease was admitted to be Asiatic cholera, and of course was imported in some way, we may have to adopt Prof. Austin Flint's opinion ("Practice of Medicine," fourth edition, p. 499): "There can be little or no doubt that the special cause" (of cholera) "may be transported in clothing and other substances after the manner of fomites. In other words, the disease is portable, without being contagious or infectious." As the first fatal case (the initial cases are not always fatal) occurred on February 9th, we must suspect vessels from Hamburg, Bremen, Port Simon, or Liverpool. As the outbreak commenced with twelve cases in the week ending April 6th, we may suspect vessels from Hamburg, Liverpool, Palermo, Bremen, or Mexico, as bringing the fomites of the disease. As there was more cholera in Hamburg last year than in any other of these places, that is the most probable starting point, although many emigrants from Central Europe, where cholera was prevailing, also came to Bremen and perhaps to Palermo.

The above facts accord with the mode of introduction of cholera into New Orleans in 1848, when vessels from Hamburg, Bremen, and Havre, brought the disease, and the ship Swanton from Havre became infected by German immigrants before there was any cholera in Havre. At that time chests of infected clothing from Pesth, in Hungary, were more than suspected, almost proved, to have brought the disease. According to the latest accounts, there have been no less than one hundred and four thousand deaths from cholera in Hungary this year, up to Sept. 1, 1873, and there were many thousands in 1872, not only in Hungary, but in Poland, Prussia, and notably in the cities of Warsaw, Konigsberg, Elbing, Dantzic, Stettin, Dresden, Leipsic, Hamburg, and other places near the Baltic, and North Sea, or German Ocean.

It is very singular that cholera did not seem to spread down into Texas this year, but appeared to be carried by rail and steamboat to Mobile, Memphis, Nashville, Cincinnati, and many other places.

In Nashville, the mortuary lists are kept by the undertakers only; no cases are reported during life, and generally only twenty-four or more hours after death. Of the first one hundred fatal cases, only twelve or fifteen have been hunted up. According to Dr. Bowling, editor of the *Nashville Journal of Medicine and Surgery*, August 1873, No. 1, p. 87, Mary Payne, colored, who lived near Wilson's Spring Branch, was the first fatal case; she seems to have been a washer-woman, as she *washed* all day on Wednesday, May 28th, and then was seized at night and died in eight hours. Mrs. Patterson, who had been with Mary Payne, was the next victim. J. McKisic, who had been with Mrs. Patterson in her sickness, and lived near her, was the third; and a colored man at the same house, was the fourth fatal case in succession. Mrs. Murray and family moved away from Wilson's Spring Branch after the death of Mary Payne, and a few days after she and her two sons occupied the same grave. Here are seven correlated cases in succession. The negro villages on the highlands and lowlands outside of the city suffered most severely. Dr. Poynor describes these villages as dense aggregations of little huts, with eight, ten, and frequently more persons crowded into one miserable little hovel. Any cleanly and sanitary precautions about these huts were never dreamed of. Excrement of every character was deposited in, between, and around the shanties. The mortality, of course, was very large, while the cleanest and best parts of Nashville generally escaped, as did Edgefield, a large town just across the Cumberland river.

The panic in Nashville was very great, and fugitives spread the disease all over the country, especially to the railroad stations to the north.

The outbreak in Franklin, Kentucky, has been best observed by Dr. Charles N. Edwards, of that place. He says: "The first well-marked case was that of Mr. H., a citizen of Franklin, who had been employed in Gallatin, Tenn. (just north of Nashville), while cholera prevailed there. He was brought home sick with cholera on June 14th, and was attended by Dr. F. The first fatal case was Mr. G. R., who had also been in Gallatin. Dr. F. was the third case, but recovered; next his child died of cholera; then his washer-woman was, at a distant house, seized, but recovered; then her daughter, who lived with her, died, and her two children."

Louisville escaped, as usual. In Cincinnati, a lady arrived about June 1st, from Nashville, nearly died of cholera, and then went on to Wheeling, West Virginia. One or more infected steamboats arrived from New Orleans and Memphis, and some of the most distinguished physicians of Cincinnati, those of the highest social and professional standing, informed me that cases could be traced to these vessels. In some of the hospitals which I visited, no record was kept of the names of the boats, streets, or numbers of the houses, from which the cases came. The first fairly-recognized fatal case occurred on June 14. The majority of the well-educated physicians of Cincinnati regarded the disease as true imported Asiatic cholera. The newspapers and health authorities regarded it as indigenous, as purely local in its character, and caused principally by imprudencies in diet. It was not regarded as communicable in any way; none but fatal cases were reported, and then only from one to two days after death. The city authorities gave very little assistance to physicians in their struggle with the disease. Disinfectants were very imperfectly employed. The general sanitary measures were reasonably good. Many of the fatal cases were reported in the newspapers, after June 14th, with name, residence, and duration of attack. The highest number of reported deaths on one day was seventeen, on June 28th. Then the disease persisted obstinately as a pure house epidemic nearly to September. There were many mild cases, and from two to four or more fatal cases a day. It spread from families to friends, until the disease was finally scattered or sprinkled thinly all over the city.

Cholera lingered in Cincinnati longer than in any other Northern city. And it always seemed, if Dr. Budd, of Bristol, and his efficient aid, Dr. Davies, had been there, the epidemic could have been stamped out in a week or two. In Dayton, Columbus, Indianapolis, Pittsburgh, Wheeling, Chicago, and other places, active sanitary measures quickly checked the outbreaks.

Chattanooga, Lebanon, and other places, received the disease from Nashville (see *Nashville Journal of Medicine and Surgery*, October, 1873, pages 193, 194 and 211). The history of cholera at Lancaster, Kentucky, described by Drs. Berry and Wilson (see *American Practitioner*, October, 1873, pages 103 to 197), is the best record of an outbreak yet published. The first case was imported from Jonesboro, Tenn., where cholera was prevailing, but did not prove fatal until two others contracted from it had succumbed. Of forty cases, only seven recovered. All were traced to communication, or to the rise of water from Tate's Well, contaminated by discharges from the first and other cases. The North-German steamers have lately brought cholera to Havre, and from there it has been carried up to Paris.—*N. Y. Med. Jour.*

